# The post-ABI effect—long-term impact of aging on participation after acquired brain injury

**DOI:** 10.1007/s00415-025-13423-3

**Published:** 2025-10-06

**Authors:** Nikki S. Thüss, Heleen M. den Hertog, Coen A. M. van Bennekom, Jacoba M. Spikman, Joukje van der Naalt

**Affiliations:** 1 Department of Neurology, Subdepartment of Neuropsychology, University Medical Center Groningen, University of Groningen, Groningen, The Netherlands; 2 Department of Neurology, University Medical Center Groningen, University of Groningen, Groningen, The Netherlands; 3 Department of Neurology, Isala Hospital Zwolle, Zwolle, The Netherlands; 4 Department of Research and Development, Heliomare Rehabilitation Center, Wijk aan Zee, The Netherlands; 5 Department of Public and Occupational Health, Amsterdam University Medical Center, Amsterdam, the Netherlands

**Keywords:** Acquired brain injury, Aging, Participation, Cognition, Psychological factors

## Abstract

**Introduction:**

Patients with a history of acute Acquired Brain Injury (ABI), including traumatic brain injury and stroke, will encounter the consequences of aging and its concomitant cognitive decline, which might result in a decline of participation level. This study aimed to assess the long-term clinical profile and participation level course in a large cohort of aging ABI patients.

**Methods:**

Multi-center observational study (BRAIN-ReADAPT study), recruiting 550 patients aged 50–68 years, at least five years post-ABI. Current participation levels were compared to levels post-ABI to assess the participation level course. Cognitive complaints and psychological factors were measured by validated questionnaires. With group comparisons and regression analyses the influence of demographic and injury-related factors were examined.

**Results:**

A decline in participation level was reported by 43% independent of age, sex, type and severity of ABI or injury chronicity. Patients experiencing this decline reported more cognitive complaints (especially mental slowness, memory, concentration difficulties) and experienced higher levels of fatigue and emotional distress, with lower resilience. No difference in current participation level was present between groups, although more restrictions and less satisfaction with participation were reported when a decline in participation was present.

**Conclusions:**

A substantial proportion of aging ABI patients experience a secondary decline in participation. This phenomenon is referred to as the *post-ABI effect*. This effect is not associated with demographic or injury-related characteristics, but resilience might be a mitigating factor. Greater awareness in clinical practice of this post-ABI effect is needed to identify potential targets for treatment to cope with this effect.

## Introduction

Acute Acquired Brain Injury (ABI), including Traumatic Brain Injury (TBI) and stroke, is a common condition [1]. As demographic trends shift towards an aging population, the incidence of acute ABI is expected to increase [2, 3]. As a result, a growing number of individuals will experience acute brain injury at some point in their lives and face its long-term consequences, which may include impairments in physical, emotional, behavioral, and cognitive functioning—often leading to reduced societal participation [4, 5]. Nevertheless, after this initial decline in participation level, most patients are able to recover to a certain extent and resume pre-injury activities with or without adaptations. Over time, these patients will be confronted with the consequences of normal aging, recognized for its concomitant decline in cognitive functions. Hence, the question arises to what extent this leads to an additive or even synergetic effect of the previously sustained ABI-related impairments.

Recovery during the first few weeks and months after an acute ABI is typically characterized by progressive improvement in cognitive functions, but its course varies depending on the severity of the injury. Complete recovery of cognitive functions is expected in most mild TBI cases within three months post-injury [6]. Similarly, most patients recover after a Transient Ischemic Attack (TIA), with cognitive impairment found in only a minority of these patients [7, 8]. In more severe cases of acute ABI, including both TBI and strokes, cognitive impairments are more common and persisting. Some improvement can still occur beyond one year after injury, but in general not up to premorbid levels [9, 10]. Hence, the sequelae of severe ABI are considered to be chronic. This can lead to reduced ability to return to work and a decline in participation level in the community, irrespective of the type of ABI [5, 11]. In addition to these cognitive and functional challenges, psychological factors such as anxiety and depression are known to influence recovery and long-term outcomes after acute ABI [12], potentially exacerbating difficulties in maintaining participation level over time. Although short-term recovery trajectories after ABI are well described, there is a knowledge gap on how aging and time since injury interact in the longer term, particularly with regard to participation outcomes.

With advancing age, there is a gradual decline in cognitive functions, in particular information processing capacity, memory and executive functioning, [13]. Despite this age-related decline in cognition, healthy older people typically maintain a high level of participation [13]. One explanation could be the role of compensatory mechanisms [14–16]. Similarly, these compensatory mechanisms may help to reduce the cognitive deficits associated with brain injuries [14, 17, 18]. In individuals with a history of acute ABI, these compensatory mechanisms might already be compromised when encountering the cognitive decline related to aging [19]. Consequently, aging ABI patients may find it harder to compensate for the additional cognitive decline, potentially resulting in a secondary decline in participation level. An initial step to substantiate this hypothesis is to map the experienced course of participation level in a large group of aging ABI patients.

Previous research on the combined effects of acute ABI and concomitant aging processes on cognition is scarce. One study showed that the rate of cognitive decline as a result of TBI and aging is similar to the rate of decline in healthy aging [20]. This suggests no accelerated rate of decline in aging ABI patients, but consequences for participation were not investigated. Other studies in TBI and stroke have shown that both longer injury chronicity and older age at the time of ABI were related to lower levels of participation level and outcome several years post-injury [21–23]. However, these studies primarily focused on participation as an outcome variable, without comparing it to prior participation levels. Given the limited research on the combined effects of a previous acute ABI and subsequent aging on participation, along with the potential impact of psychological factors such as anxiety and depression, further exploration is warranted.

This study aims to investigate the clinical presentation and perceived long-term course of participation in patients years after an acute ABI. Additionally, we will explore the complaints associated with perceived participation levels, as well as the influence of demographic, injury-related, and psychological factors.

## Methods

### Study design and participants

The BRAin INjury REcovery And Disability in Aging PaTients (BRAIN-ReADAPT) study is an observational multi-center cohort study conducted in the Netherlands. Participants were recruited from two trauma centers and two rehabilitation clinics. Patients were identified through hospital databases and screened for eligibility based on information from their medical records. Eligible individuals were then contacted by letter from their local healthcare provider. This approach was used because an existing patient-care provider relationship was required prior to contacting potential participants. One reminder letter was sent to enhance response rates. Recruitment took place between March 2021 and January 2024. The following inclusion criteria were used: (a) current age between 50 and 67 years, rationale: enables the patient to experience the effects of aging with a low risk of neurodegenerative disease; (b) diagnosis of acute ABI (traumatic brain injury, stroke or subarachnoid hemorrhage (after the age of 25 years); (c) having sustained the ABI more than 5 years ago; (d) able to independently fill out questionnaires. Exclusion criteria were: (a) major psychiatric disease for which the patient is currently treated; (b) severe comorbidities (c) neurological or neurodegenerative disease; (d) substance abuse; (e) insufficient comprehension of the Dutch language. Patients who either did not return the questionnaire or provided incomplete answers were excluded from the analysis. Severity of ABI was classified as follows: TBI was defined based on the Glasgow Coma Scale sum score 13–15 = mild, 9–12 = moderate and 3–8 = severe. Stroke was categorized in Transient Ischemic Attack (TIA), which is considered ‘mild stroke’ without Computed Tomography (CT) -abnormalities, intracerebral hemorrhage, subarachnoid hemorrhage and ischemic stroke. This study was executed in accordance with the Declaration of Helsinki (2013) and was approved by the University Medical Center Groningen Medical Ethics Committee (NL79072.042.21). All participants gave written consent prior to participation.

### Procedure

After providing informed consent, validated questionnaires were administered by mail or online. Demographics, injury-related characteristics (including intracranial injury on a CT- or Magnetic Resonance Imaging (MRI)- scan assessed by a certified radiologist) and medical history were obtained from medical records. Medical history included the presence of multiple ABI, mental health issues (for which the patient was treated in the past), and comorbidities, including cardiovascular disease, diabetes, respiratory disease, malignancy and dyslipidemia as determined at the time of index injury, which was comprised for the current study to a binary variable (comorbidities, present or absent). The chain of care following the injury, including hospital admission, discharge destination was assessed through self-report.

### Questionnaires

#### Participation level and outcome

**Fig. 1:**
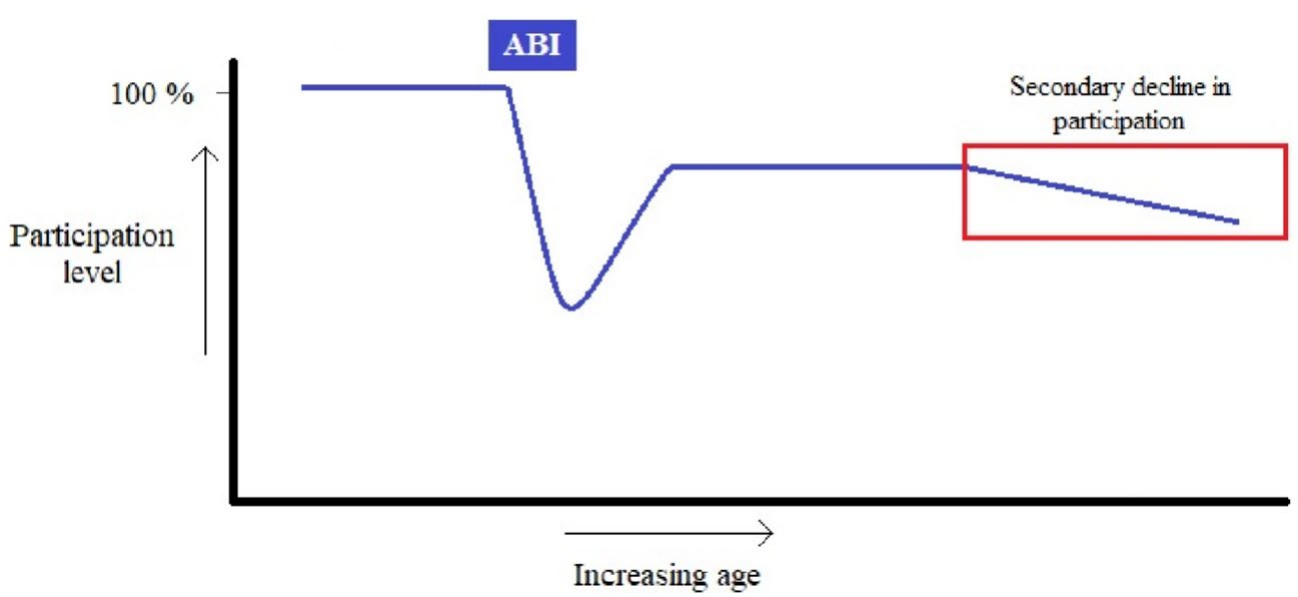
Secondary decline in participation

*Self-rated course of participation level.* Patients were asked to rate retrospectively (1) their maximum level of functioning after the initial recovery period post-ABI with a percentage from 0 to 100 and (2) their current level of functioning. This hypothesized secondary decline in participation level, as shown in Fig. 1, is defined by a negative difference between the maximum level and the current level of functioning. Scores were dichotomized into two categories: presence or absence of a secondary decline in participation level (decline in participation and no-decline in participation). For patients missing this score (*N* = 23), a decline was determined using the reports of a significant other on a validated questionnaire assessing decline of functioning in daily life with a cut-off score of 3.31 as recommended by the author [24, 25].

*Utrecht Scale for Evaluation of Rehabilitation-Participation (USER-P)* [26]. The USER-P assesses the current participation level and distinguishes between 3 subscales. The first subscale “frequency of participation” describes the amount of vocational participation, leisure and social activities and thereby objectifies the level of participation. The subscale: “restrictions”, measures the level of perceived limitations (a higher score meaning less restrictions) and the last subscale “satisfaction”, assesses the level of satisfaction in participation. A total score is derived from each subscale on a 0–100 scale, with higher scores indicating a higher level of participation.

*Changes in cognitive functioning reported by significant other—Informant Questionnaire on Cognitive Decline in Elderly (IQCODE)* [25]*.* Significant others report once, at the time of participation in the current study, on changes noticed in the past years in cognitive functioning in daily life tasks for the acute-ABI patients in 16 questions with scores ranging from 1 to 5. The sum of these scores is divided by the total number of questions, with higher scores indicating a more severe decline.

*Quality of life – Quality of Life after Brain Injury-Overall Scale (QOLIBRI-OS)* [27] assesses health-related quality of life in domains most often affected by brain injury with 6 items. Answers are rated from 1 (not at all) to 5 (very), resulting in a sum score ranging from 0 to 30. The sum score is arithmetically converted to a percentage (0 to 100) with 0 representing the lowest QoL and 100 the highest.

#### Complaints and psychological characteristics

*Complaints—Checklist Cognition and Emotion-24 (CLCE-24)* [28] is used only to assess the presence of cognitive complaints (13 questions) with a yes/no answer (range 0–13). Presence of additional often-reported complaints, such as headache and sensitivity to noise were assessed with separate questions.

*Fatigue—Dutch Multifactorial fatigue scale (DMFS)* [29] measures various aspects of fatigue, including mental fatigue (DMFS-M, 7 items) and physical fatigue (DFMS-Ph, 6 items), which were included in the current study. Answers are rated on a 5-point Likert scale (1 = totally disagree to 5 = totally agree). Lower scores are indicative for less fatigue.

*Emotional distress—Hospital Anxiety and Depression Scale (HADS)* [30] contains 7 items measuring anxiety (HADS-A) and 7 items which measure depression (HADS-D). Scores range from 0 to 21 per subscale, higher scores are indicative for more emotional distress.

*Social support—Social Support List—Interaction-12 (SSL-I-12)* [31] assesses the amount of social support. A total of 12 items (scored 1–4) results in a sum score ranging from 12 to 48. A higher score is indicative of more social support.

*Resilience—Resilience Evaluation Scale (RES)* [32] measures psychological resilience with 9 items, with responses ranging from 1 (completely disagree) to 5 (completely agree). The sum of these responses results in a total score (range 5–25), with a higher score indicating greater psychological resilience.

### Statistical analysis

Data analyses were conducted using Statistical Package for the Social Sciences (version 28.0). Demographics, injury-related factors and psychological characteristics were described for the total cohort, and divided into two groups: patients who experienced a decline in participation level and those who did not (Students’ *t*-test) and non-parametric (Mann–Whitney *U* test) measures were used to summarize differences in means between the two groups. Differences in categorical variables were calculated with Chi-squared tests. Spearman’s correlations were calculated to examine the associations between anxiety, depression, and levels of mental and physical fatigue. A binary logistic regression analysis was conducted on the total sample to examine the influence of types of ABI (TBI, hemorrhagic stroke including subarachnoid hemorrhage (SAH) and ischemic stroke including TIA) on the likelihood of experiencing a decline in participation level adjusting for sex, age at time of injury and injury chronicity. After verifying assumptions of normality and homogeneity of variances, a one-way ANOVA was conducted to examine differences between ABI groups on the USER-P scales. ANCOVAs were performed to determine whether group effects remained after controlling for age. Tukey post-hoc tests were conducted to examine pairwise differences between ABI groups. Kruskal–Wallis *H*-test was used to compare three ABI groups for a variable without normal distribution. Missing values did not exceed 5%, except for discharge destination (16%). For all other analyses, an *α* level of 0.05 was used. Data and materials used in this study are not publicly available due to ethical and privacy considerations but may be made available upon reasonable request to the corresponding author.

## Results

### Study population characteristics

**Fig. 2:**
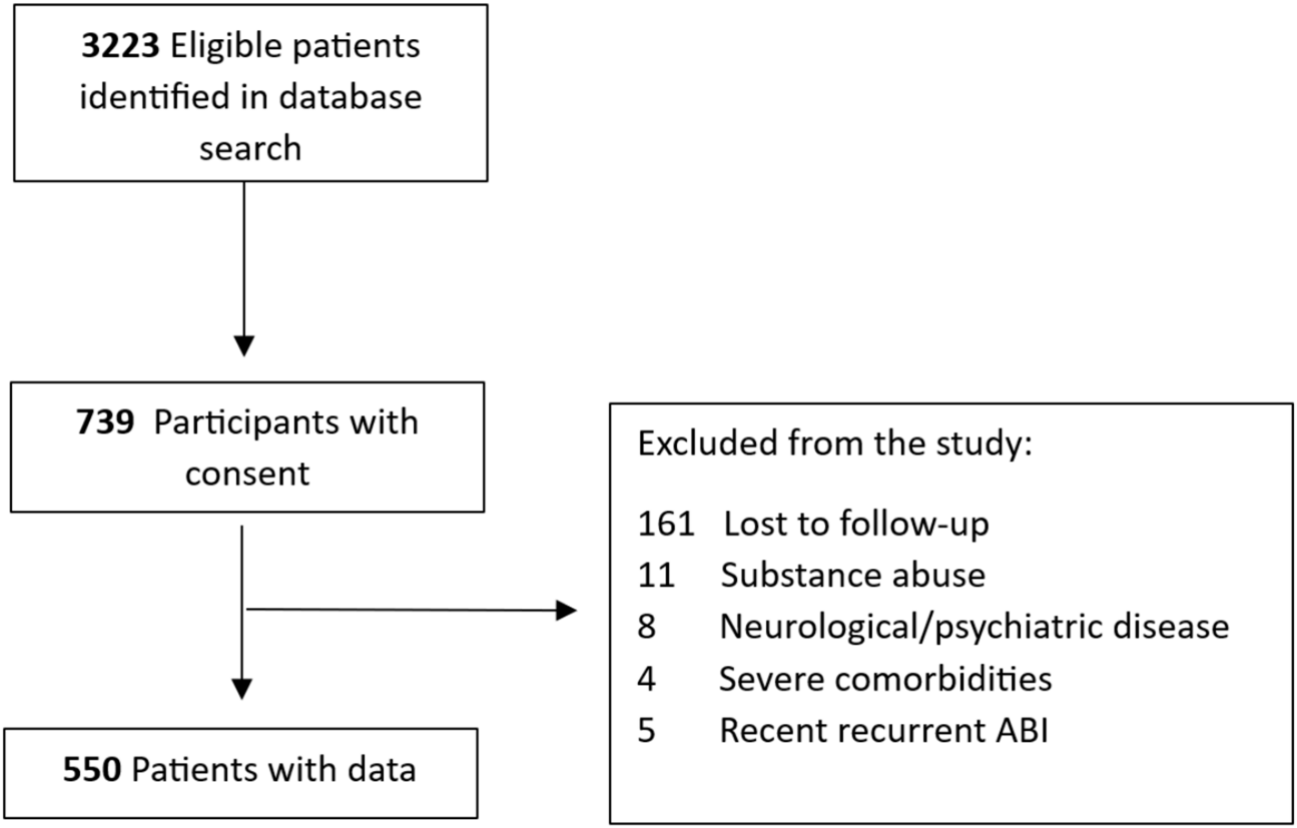
Flow diagram of inclusion

We identified a total of 3223 eligible patients, of whom 739 patients provided informed consent. Data from 550 patients were analyzed in the current study (see Fig. 2). There were no differences in age (*U* = 56,368.50, *p* = 0.440) or sex (*χ*^2^ = 0.016, *p* = 0.899) between patients who were included and those who did not participate. Injury chronicity defined as the years between the index brain injury and reference date did differ significantly (*U *= 48,664.00, *p* = 0.024); patients who participated had a longer timespan (*M* = 12.72, SD = 4.51) than those who were excluded (*M* = 11.73, SD = 3.63). Patients with hemorrhagic stroke (28%) and ischemic stroke (34%) had more comorbidities compared to TBI patients (14%) (hemorrhagic stroke: *χ*^2^ = 8.872, *p* = 0.003; ischemic stroke: *χ*^2^ = 21.397, *p* < 0.001).

Demographic and injury-related factors of the participants are summarized in Table 1. Current age ranged between 50 and 68 years. Questionnaires were received on average 10.7 years after the index brain injury (SD = 4.42).

**Table 1:** Demographic variables in the total acute-ABI patient group divided in the patients that experienced a secondary decline in participation level (decline) and those who did not (no-decline)

	Total (*n* = 550)	Declined (*n* = 237)	No-decline (*n* = 313)	*p*-value
Age, at time of participation	60.5 ± 4.9	60.2 ± 5.0	60.7 ± 4.8	.313^1^
Sex, female	270 (49)	123 (52)	147 (47)	.252^2^
Education				.641^2^
Low (1–3)	15 (3)	6 (3)	9 (3)	
Medium (4–5)	300 (55)	135 (57)	165 (53)	
High (6–7)	229 (42)	94 (40)	135 (44)	
Living situation				.837^2^
Alone	74 (14)	33 (14)	41 (13)	
With others	472 (86)	204 (86)	264 (87)	
SSL-I-12	29.76 (6.45)	29.83 (6.74)	29.71 (6.24)	.664^1^
Employment status				
Employed	269 (49)	113 (48)	156 (50)	.654^2^
Unable to work	142 (26)	57 (24)	85 (27)	
(early) retirement	91 (17)	44 (19)	47 (15)	
Voluntary work	34 (6)	16 (7)	18 (6)	
Seeking employment	9 (2)	5 (2)	4 (1)	
Comorbidities, yes	139 (25)	66 (28)	73 (23)	.227^2^
Mental health problems, yes	25 (5)	10 (4)	15 (5)	.749^2^

*P*-values are for differences between groups

Results are given as n (%) or M ± SD

*SSL-I-12* Social Support List-Interactions-12. Educational level with Verhage scale

*Significant at *p* < 0.05

^a^Mann-Whitney-*U*

^b^Chi-kwadraat

### Participation level

**Fig. 3:**
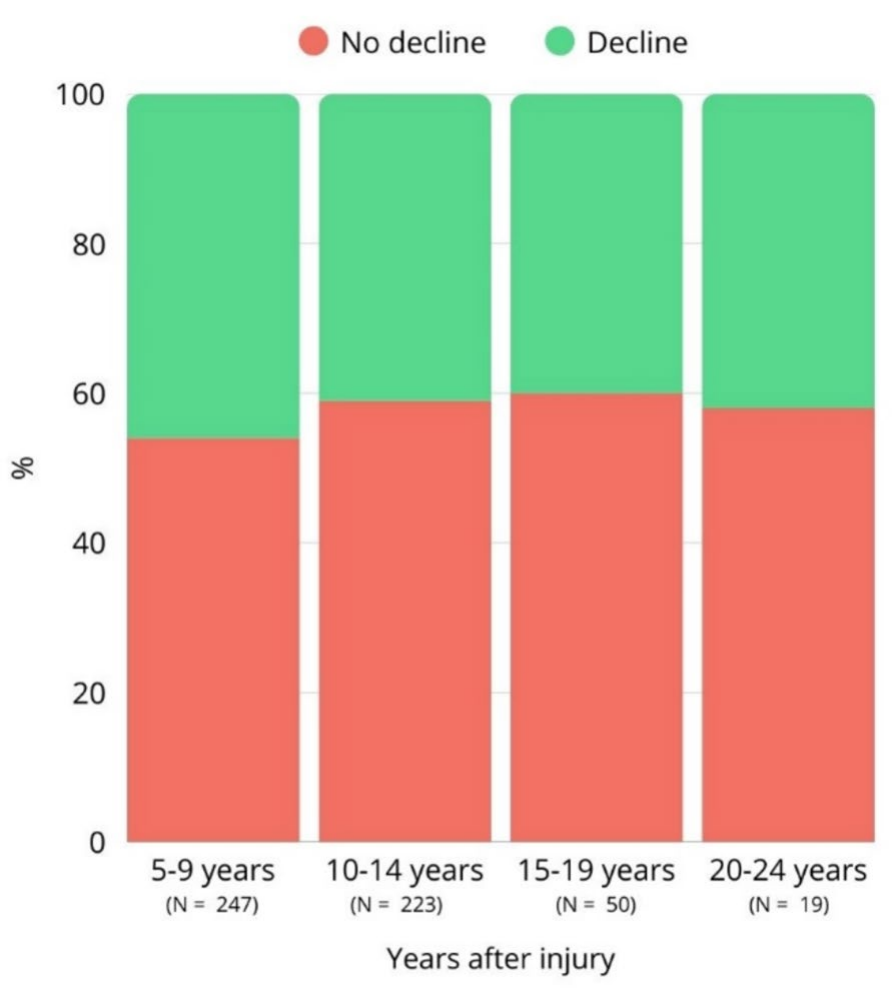
Proportion of patients experiencing a secondary decline in participation level (decline) and those who did not (no decline) across injury chronicity groups (5-year intervals)

A decline in participation level was reported by 43% (N = 237) of the patients. No significant differences were found between patients who did and those who did not experience a decline in participation level regarding demographic factors such as sex and age, or injury-related factors (Tables 1 and 2). Figure 3 illustrates the proportion of patients experiencing a decline in participation level across injury chronicity groups with 5-year intervals. Due to insufficient data, groups with ≥ 25 years post-injury were not included in this figure. When comparing patients with a decline in participation level to responses from their significant others (*N* = 410), significant others reported higher scores on the IQCODE in the group with a decline in participation compared to no decline (Table 3). This suggests that significant others perceived greater cognitive decline in patients who also reported a decline in participation level. Significant others correctly identified in 67.3% (113/168) the presence of a decline and in 49.3% (110/223) the absence of a decline, indicating moderate accuracy in recognizing this.

**Table 2:** Injury-related factors and path of care after injury in the total acute-ABI patient group and divided into the patients that experienced a secondary decline in participation level (decline) and those who did not (no-decline)

	Total (*n* = 550)	Decline (*n* = 237)	No-decline (*n* = 313)	*p*-value
Multiple ABI, yes	22 (4)	10 (5)	12 (4)	.882^b^
ABI Type				.976^b^
TBI	196 (36)	83 (35)	113 (36)	.207^b^
Mild	142 (77)	65 (81)	77 (73)	
Moderate to severe	43 (23)	15 (19)	28 (26)	
Stroke type				.450^b^
*Ischemic*				
Transient ischemic attack	85 (25)	42 (29)	43 (23)	
Ischemic stroke	140 (42)	61 (43)	79 (42)	
*Hemorrhagic*				
Subarachnoid hemorrhage	58 (18)	21 (15)	37 (20)	
Intracerebral hemorrhage	47 (14)	19 (13)	28 (15)	
Intracranial injury, yes	332 (65)	141 (63)	191 (67)	.367^b^
Injury chronicity, years	10.7 ± 4.4	10.5 ± 4.2	11 ± 4.6	.205^a^
Care after injury				
Hospitalized	463 (85)	197 (85)	266 (86)	.976^b^
Discharged from ED	59 (11)	25 (11)	34 (11)	
Non-hospitalized	20 (4)	9 (4)	11 (4)	
Discharge destination				.064^b^
Home	303 (66)	138 (71)	165 (63)	
Rehabilitation or care facility	156 (34)	57 (29)	99 (38)	
Care after discharge from hospital, yes	316 (58)	143 (61)	173 (56)	.214^b^
Follow-up at hospital	160 (29)	72 (30)	88 (28)	
Occupational physician	67 (12)	32 (14)	35 (11)	
General practitioner	46 (8)	21 (9)	25 (8)	
Psychologist	130 (24)	55 (23)	75 (24)	
Physiotherapist	184 (33)	77 (33)	107 (34)	
Rehabilitation physician	203 (37)	93 (39)	110 (35)	

*P*-values are for differences between groups

Results are given as *n* (%) or M ± SD

*ABI* Acquired Brain Injury, *ED* Emergency Department, *SAH* Subarachnoid Hemorrhage, *TBI* Traumatic Brain Injury

*Significant at *p* < 0.05

^a^Mann-Whitney-*U*

^b^Chi-kwadraat

**Table 3:** Participation level and outcome of total, divided in the patients that experienced a secondary decline in participation level (decline) and those who did not (no-decline)

	Total (*n* = 550)	Decline (*n* = 237)	No-decline (*n* = 313)	*p*-value
User-p				
Frequency	32.41 (10.17)	31.83 (9.21)	32.84 (10.82)	.195^a^
Restrictions	83.65 (17.49)	81.76 (18.29)	85.07 (16.77)	.016*^a^
Satisfaction	70.67 (16.35)	67.86 (16.37)	72.83 (16.04)	< .001*^a^
QOLIBRI-OS	68.53 (16.21)	64.77 (15.80)	71.43 (15.95)	< .001*^a^
IQCODE	3.15 (0.48)	3.22 (0.49)	3.09 (0.46)	< .001*^a^

Results are given as M (SD)

*IQCODE* Informant Questionnaire on Cognitive Decline in Elderly, *QOLIBRI-OS* Quality of Life after Brain Injury-Overall Scale, *USER-P* Utrecht Scale for Evaluation of Rehabilitation-Participation

*Significant at *p* < 0.05

^a^Mann-Whitney-*U*

Table 3 shows the current level of participation for the total group, along with the group differences for decline in participation level or no decline. No significant differences were found between the groups on the USER-P frequency of participation. However, the group who experienced a decline reported lower scores on the USER-P restrictions and satisfaction scales. Distribution of employment status did not differ between the group that experienced a decline in participation level and those who did not. Men did not demonstrate a significantly higher rate of paid employment compared to women (53% vs. 45%; *p* = 0.059) but scored higher on the USER-P restriction scale (*p* = 0.002).

### Complaints

On average, patients reported 5 cognitive complaints, ranging from 0 to 13. The most frequently reported cognitive complaints were mental slowness (reported by 71%), memory problems (reported by 67%) and concentration difficulties (reported by 62%). No differences were found between men and women in the number of reported cognitive complaints. However, women experienced more often sensitivity to noise (reported by 73%, *p* = 0.001) and complaints of headache (reported by 36%, *p* = 0.032) compared to men.

The group with a decline in participation reported significantly more complaints compared to the group with no decline, on average 6 cognitive (range 0–13) compared to 4 (range 0–13). Also, patients who experienced a decline were significantly more sensitive to noise and experienced more often headaches.

### Injury type

Patients with TIA had a paid employment rate of 44%, those with an SAH 41%, intracerebral hemorrhage 57%, and ischemic stroke 53%. In TBI patients, rates were 67% for mild and 51% for moderate to severe TBI. Age differed among the ABI groups (*F* = 6.51, *p* = 0.002): TBI patients were significantly younger (Mean Age = 59.5, SD = 4.89) than patients with ischemic stroke (Mean age = 61.2, SD = 4.63) (*p* = 0.001).

Binary logistic regression, adjusted for sex, age at time of injury, and injury chronicity, did not show that type of ABI was an independent factor associated with experiencing a decline in participation level.

Post-hoc analysis (Table 4) showed that TBI patients scored higher than ischemic stroke patients regarding the current participation level. Similarly, differences were observed between all groups on the USER-P restriction scale, with ischemic stroke patients showing the lowest scores. TBI patients scored higher on the USER-P satisfaction scale compared to ischemic stroke patients. Group effects on all USER-P scales remained significant after adjusting for age. Regarding the presence of intracranial injury, no differences were found on the USER-P frequency of participation and satisfaction scales between patient groups, although patients with intracranial injury reported significantly lower scores on the USER-P restrictions (*p* = 0.022), indicating that they perceived more restrictions in participation.

**Table 4:** Differences in USER-P scores across ABI groups

	Ischemic stroke	Hemorrhagic stroke	TBI	Statistical test	*p*-value
User-p					
Frequency	31.14 (10.10)	31.93 (10.37)	34.28 (9.90)	F(2, 521) = 5.071	.007*
Restrictions	80.99 (17.94)	79.84 (18.78)	89.43 (14.49)	H(2) = 32.201	< .001*
Satisfaction	68.55 (15.26)	71.43 (17.82)	72.97 (16.09)	F(2, 522) = 3.896	.021*

Results are given as M (SD)

*ABI* Acquired Brain Injury, *SAH* Subarachnoid Hemorrhage; *TBI* Traumatic Brain Injury, *USER-P* Utrecht Scale for Evaluation of Rehabilitation-Participation

*Significant at *p* < 0.05

### Psychological factors and quality of life

The groups (decline in participation or not) were also compared on several psychological factors. Table 5 shows differences across all psychological factors. Patients with a decline in participation level reported higher scores on fatigue (physical and mental), emotional distress, and lower scores on resilience. Spearman’s correlations between anxiety, depression, and both mental and physical fatigue showed positive moderate to moderately strong correlations (*r*s = 0.495 for depression and mental fatigue, *r*s = 0.552 for depression and physical fatigue, *r*s = 0.442 for anxiety and mental fatigue, and *r*s = 0.451 for anxiety and physical fatigue; all *p* < 0.001).

**Table 5:** Overview of psychological characteristics of total group, and divided into the patients that experienced a secondary decline in participation level (decline) and those who did not (no-decline)

	Total (*n* = 550)	Decline (*n* = 237)	No-decline (*n* = 313)	*p*-value
Cognitive complaints	4.83 (3.39)	5.54 (3.15)	4.30 (3.47)	< .001*^a^
Headache	172 (32)	86 (37)	86 (28)	.027*^b^
Sensitivity to noise	363 (66)	174 (74)	189 (61)	.001*^b^
DMFS-M	24.73 (6.73)	25.85 (6.24)	23.86 (6.97)	.001*^a^
DMFS-Ph	17.48 (5.35)	18.72 (5.30)	16.52 (5.20)	< .001*^a^
HADS-A	5.34 (4.05)	6.07 (4.27)	4.77 (3.78)	< .001*^a^
HADS-D	4.54 (3.89)	5.03 (3.77)	4.17 (3.94)	< .001*^a^
RES	23.62 (6.88)	22.63 (6.93)	24.39 (6.76)	.005*^a^

Results are given as *n* (%) or M (SD)

*DMFS-M* Dutch Multifactorial fatigue scale-Mental, *DMFS-Ph* Dutch Multifactorial fatigue scale-Physical, *HADS-A* Hospital Anxiety and Depression scale-Anxiety, *HADS-D* Hospital Anxiety and Depression scale-Depression, *RES* Resilience Evaluation Scale

*Significant at p < 0.05

^a^Mann-Whitney-*U*

^b^Chi-kwadraat

Patients with a decline in participation level also reported a lower score on the QOLIBRI than patients with no decline. Scores on the QOLIBRI were not different between men (*M* = 20.68, SD = 4.73) and women (*M* = 20.43, SD = 5.00) (*p* = 0.559). Also no differences between the ABI groups, *F*(2, 535) = 2.03, *p* = 0.133 were present. Mean scores were 21.11 (SD = 4.83) for TBI, 20.15 (SD = 4.67) for ischemic stroke, and 20.55 (SD = 5.20) for hemorrhagic stroke.

## Discussion

In this study, we showed that nearly half of the patients with acute ABI experienced a secondary decline in participation level, years after the initial injury. We refer to this as the *post-ABI effect*. In many cases, the post-ABI effect was supported by reports from their significant others. Mental slowness, memory problems and concentration difficulties were the most commonly reported cognitive complaints.

The post-ABI effect was reported by 43% of patient, without observed sex differences for this effect. In general, participation difficulties in the chronic phase after acute ABI are well-documented in previous research [33]. However, the post-ABI effect appeared to be unrelated to the current level of vocational or leisure participation. This is further supported by the finding that employment status was comparable between patients who experienced the post-ABI effect, and those who did not. Despite similar participation outcomes, patients who experienced the post-ABI effect encountered more restrictions and were less satisfied with their participation level. This discrepancy may be explained by patients continuing their usual activities at the cost of more cognitive effort that might result in mental fatigue. In this context, the increase of restrictions and less satisfaction might reflect the perceived burden or difficulty of maintaining a comparable level of participation. In the present study, patients with the post-ABI effect also experienced higher levels of mental fatigue. Especially mental fatigue can result from cognitive impairments after TBI or stroke, as individuals need more effort to process information and manage everyday tasks [34]. The fact that mental slowness was one of the most reported cognitive problems underlines this reasoning. We realize that no specific data were obtained on whether patients had already reduced their workload or participation levels prior to the reported decline. Monitoring participation levels over time would therefore be an important future direction to better understand the post-ABI effect and provide insights into whether early reductions in workload may help mitigate or delay the post-ABI effect.

Even though cognitive complaints may partially reflect objective cognitive impairments due to brain injury, they are also commonly associated with other factors such as emotional distress [35]. Previous research has shown that emotional distress is common in the chronic phase after ABI and can be associated with difficulties in participation [36, 37]. This was supported by our findings, as patients with the post-ABI effect reported significantly higher levels of depression and anxiety. Also, higher levels of emotional distress were associated with increased mental fatigue. This is consistent with the observation that fatigue can also be a consequence of ABI-related factors such as depression or anxiety [38].

Although psychological factors seem to be important contributors to the post-ABI effect, the role of demographic and injury-related variables on the occurrence of the post-ABI effect also must be considered. We did not find differences in demographic factors between patients who experienced the post-ABI effect and those who did not, suggesting that it is not influenced by factors such as sex, educational level, or age. Sex differences were not observed in the number of reported complaints, but only in the type: women reported more frequent headaches and greater sensitivity to noise compared to men. While previous research has shown that women experience more complaints and worse outcomes than men after ABI [39, 40], the current study does not support this. Notably, most studies on sex differences in outcome have focused on the first year post-injury. It is possible that these differences are more pronounced in the early phase but diminish over time, as suggested by a study assessing outcomes 10 years after ABI [12], which also reported no sex differences.

When considering injury-related variables, we did not find differences between TBI and stroke patients in experiencing the post-ABI effect, even after adjusting for sex and age. This suggests that despite their distinct pathophysiological mechanisms, both subgroups may be similarly vulnerable to aging. Interestingly, despite the lack of differences in the prevalence of the post-ABI effect between TBI and stroke patients, patients with TBI seemed to report the most favorable outcomes in their current participation level, compared to other ABI groups. They demonstrated a higher current level of participation, experienced fewer restrictions, and were more satisfied with their participation level, and these differences persisted even after adjusting for age. This difference, however, did not extend to the overall quality of life, which was similar across all ABI groups. A possible explanation for the relatively favorable participation outcomes among TBI patients could be related to differences in physical functioning, as stroke patients may experience more physical impairments that limit their participation. Other injury-related factors, such as severity and injury chronicity also did not differ between patients who did experience the post-ABI effect and those who did not. This is not in line with previous research, demonstrating that injury-related factors such as injury chronicity and age at time of injury are related to participation and long-term outcome after injury [22]. This effect was most pronounced in patients older than 75. However, post-hoc analysis in our cohort showed that the prevalence of post-ABI effect is similar across different age groups (50–59 years: 43.4%, 60–67 years: 42.9%). Possibly, the restricted age inclusion criterium limited the full evaluation of the effect of injury chronicity and future studies might consider including the oldest-old patients. Older age might amplify the impact of injury-related factors, as age-associated changes such as cognitive decline and neurodegeneration might become more pronounced. Another possible explanation for the lack of impact of injury severity is the difficulty in reliably determining injury severity in a retrospective study design. At the time, many participants sustained their ABI; standardized tools such as the NIH Stroke Scale (NIHSS) were not yet widely implemented in clinical practice [41]. As a result, retrospective assessment of severity was limited. In current clinical settings, more consistent use of such measures allows for better characterization of injury severity, which should be incorporated in future prospective studies.

In general, patients can cope with post-ABI complaints by relying on compensatory mechanisms. It was hypothesized that aging ABI patients struggle to compensate for additional age-related cognitive decline, particularly when compensatory mechanisms have already been depleted due to prior brain injury. This reasoning may explain why patients experiencing the post-ABI effect report more complaints, emotional distress and fatigue as the combined burden of injury-related and age-related decline becomes increasingly challenging to manage. In this context, compensatory behavior might be supported by resilience. Resilience is often defined as the capacity to adapt positively to (new) challenges [42]. Patients with greater resilience might be better equipped to maintain participation by using compensatory mechanisms, such as finding alternative ways to complete tasks or learning to use residual skills more effectively. In our study, patients without the post-ABI effect were found to be more resilient than those with the post-ABI effect, indicating that resilience is a mediating factor in reducing the risk of the post-ABI effect. This aligns with previous studies indicating that greater resilience is beneficial for outcomes after ABI [43–45]. Other factors contributing to compensatory behavior include, for example, cognitive reserve. While resilience appears to play a protective role, our findings did not show any differences in educational level—a common proxy for cognitive reserve [15]—between patients with and without the post-ABI effect. This could indicate that cognitive reserve, at least as measured by educational level, may not be a key factor involved in the likelihood of experiencing the post-ABI effect. However, cognitive reserve is a multidimensional concept, that extends beyond formal education. Furthermore, educational level may not adequately reflect cognitive capacities in older adults, as educational opportunities and societal roles differed substantial across generations. The complex and multifaceted nature of cognitive reserve warrants more comprehensive exploration, including other indicators such as occupational complexity and engagement in cognitively stimulating activities [46].

Our findings suggest that demographic and injury-related factors may not be the primary determinants of the post-ABI effect. Instead, psychological factors, such as resilience, appear to play a more central role in determining long-term perceived participation outcomes. The post-ABI effect seems to reflect a broader decline in overall well-being, reflected in increased frequency of cognitive complaints, perceived emotional distress and fatigue with a lower quality of life.

These insights have important implications for clinical practice. Currently, standard care typically involves follow-up of acute ABI patients for up to one year, after which the most acute symptoms have subsided. However, ideally, considering the substantial proportion of patients who experience ‘new’ or worsening problems in the chronic phase, improving accessibility to care beyond the one year after the injury would be preferable. A pilot study investigated the feasibility of a rehabilitation program starting at a mean of five years after ABI and reported promising results, with increased satisfaction in participation and fewer difficulties in daily life [47]. Based on the present findings, clinicians should be aware that a substantial number of patients with acute ABI can experience a secondary decline in participation when aging, accompanied by a variety of cognitive complaints and restrictions in participation. Therefore, it is important that healthcare professionals and occupational physicians are better informed about this issue to ensure timely recognition and appropriate support for this category of patients.

### Limitations

As this study has described the post-ABI effect for the first time, we acknowledge that certain limitations of this study should be considered. First, a certain selection bias may be present, as in this retrospective study a substantial number of patients has not responded to the invitation or declined to participate due to factors such as reallocation or severe (pre-injury) comorbidities. Nonetheless, the non-participating patient group was largely comparable to the participating group, except for a shorter injury chronicity.

Second, not all data were traceable because participants were invited based on an injury that occurred many years ago. This limited our ability to fully explore the impact of injury severity. Additionally, because patients were asked to retrospectively recall their maximum level of participation after ABI without a fixed time point, variability in interpretation may exist. We aimed to capture the subjectively perceived plateau of recovery, but recognize that prospective longitudinal assessment would allow for more precise characterization of the trajectory and timing of decline in participation. Another limitation is the age range criterion in this study, which was selected to investigate participation, specifically participation in work, with the minimal risk of the presence of neurodegenerative disease. Expanding the age range might allow for a more comprehensive understanding of participation in all domains, also in the oldest-old and younger patients. Also, a control group of aging individuals without ABI was not included. Such a group could provide additional context regarding how aging trajectories in the general population compare to those in individuals with ABI. However, the scope of the current study was to explore perceived long-term changes within a clinical ABI population using a within-person reference point. As this is the first study to define and examine the post-ABI effect, we prioritized a large, diverse ABI sample to establish this phenomenon descriptively. Furthermore, detailed information on the type and intensity of rehabilitation received was not available for most participants, which limits the interpretation of potential rehabilitation effects on long-term participation outcomes.

Additionally, while our study primarily focused on cognitive aspects of aging after ABI, it is important to acknowledge that aging can also impact other neurological functions not measured in this study, such as motor skills and coordination [48] individuals with ABI can experience physical disabilities that may influence participation [49, 50]. While this was beyond the scope of our study, future research should incorporate assessments of other neurological domains, such as motor functioning, as well as neuropsychological assessment to provide a more holistic understanding of the aging process in ABI patients.

## Conclusion

The post-ABI effect occurs in almost half of the patients with acute ABI, emerging several years after the initial injury. This secondary decline in participation level is characterized by more perceived restrictions and less satisfaction in current participation level. Patients frequently experienced cognitive complaints and higher levels of emotional distress. Demographic or injury-related factors seem not to account for the presence of the post-ABI effect. Possibly, an underlying vulnerability in resilience contributes to experiencing the post-ABI effect. Greater awareness of this effect is needed in clinical practice to better inform patients and caregivers, and to support them in the chronic phase after brain injury. Further research is necessary to identify factors that can serve as potential targets for treatment aiming to cope with the long-term effect in patients with acute ABI.

## Figures and Tables

**Fig. 4:**
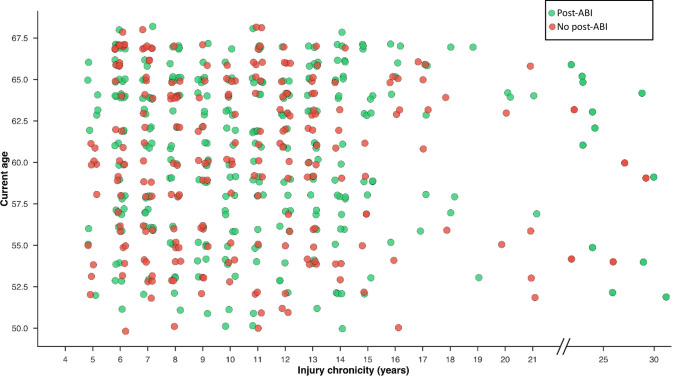
Distribution injury chronicity (years) and current age in post-ABI vs. no post-ABI effect group

## Appendix

See Fig. 4.
